# The Association between Weight Gain/Restoration and Bone Mineral Density in Adolescents with Anorexia Nervosa: A Systematic Review

**DOI:** 10.3390/nu8120769

**Published:** 2016-11-29

**Authors:** Marwan El Ghoch, Davide Gatti, Simona Calugi, Ombretta Viapiana, Paola Vittoria Bazzani, Riccardo Dalle Grave

**Affiliations:** 1 Department of Eating and Weight Disorders, Villa Garda Hospital, Garda 37016, Italy; si.calugi@gmail.com (S.C.); rdalleg@gmail.com (R.D.G.); 2 Rheumatology Unit, Department of Medicine, University of Verona, Verona 37124, Italy; davide.gatti@univr.it (D.G.); ombretta.viapiana@univr.it (O.V.); 3 Department of Radiology, Villa Garda Hospital, Garda 37016, Italy; paola.baz58@gmail.com

**Keywords:** anorexia nervosa, body composition, weight restoration, osteoporosis, osteopenia, and bone mineral density

## Abstract

Background: Reduced bone mineral density (BMD) is one of the most frequent medical complications of anorexia nervosa (AN). The purpose of this paper was to conduct a systematic review of the association between weight gain/restoration and BMD in adolescents with AN. Methods: Literature searches, study selection, method, and quality appraisal were performed independently by two authors, and data were collated using a narrative approach. Results: Of the 1156 articles retrieved, 19 studies met the inclusion criteria, and their analysis revealed four main findings. First, six studies reported that weight gain and restoration are associated with BMD stabilization after one year of follow-up from baseline. Second, seven studies with longer follow-up periods (≈16 months) reported significant improvements in BMD measures. Third, one study showed that normalization of BMD can be achieved after ≈30-month follow-up of normal-weight maintenance. Fourth, another study showed that male adolescents with AN who achieve weight gain but remain underweight may experience further BMD loss, unlike their weight-restored counterparts (BMI ≥ 19 kg/m^2^), who show a significant increase in BMD and bone mineral accrual rates that double those of healthy male adolescents. The first two findings can be considered robust, as they are supported by strong evidence. The third and fourth findings, however, derive from single studies and therefore require further confirmation. Conclusion: The literature supports weight gain as an effective strategy for promoting BMD increase in adolescents with AN. However, this process is slow, and improvements do not become detectable until ≈16-month follow-up.

## 1. Introduction

Reduced bone mineral density (BMD) is one of the most frequent medical complications of anorexia nervosa (AN) [1,2,3]. Available data indicate that nearly 85% of females with AN have very low BMD [4,5], and consequently, a seven-fold increase in the risk of spontaneous fractures with respect to healthy controls [6,7]. Not only females with AN suffer from osteopenia and osteoporosis [8,9,10], they also seem to occur in males with AN [11].

Several factors have been suggested to play a role in BMD reduction in AN. These include a very low body weight, associated with depleted lean and fat masses; estrogen or testosterone deficiencies; excessive physical activity; low levels of dependent trophic factors, in particular, IGF-1; growth hormone and ghrelin resistance; reductions in leptin, insulin and oxytocin; and increased levels of cortisol, adiponectin, and peptide YY [12].

AN typically begins during adolescence [13], a period in which bone mass increases dramatically, peaking in young adulthood at the approximate age of 25–30 [14]. By interfering negatively with the normal bone mineral accretion [15], AN in adolescents can lead to a reduction in both trabecular and cortical BMD within a few months (~6 months) [16,17]. It is therefore vital to find effective therapeutic strategies for restoring lost bone mass as soon as possible in adolescents recovering from AN. Unfortunately, most treatments tested so far, for example hormone replacement [18,19], the oral contraceptive pill [20], dehydroepiandrosterone (DHEA) [21], and biphosphonates [22,23], have resulted in only modest improvements in BMD at best in patients with AN.

In recent years, a considerable body of research has been amassed on bone status in AN, focusing specifically on the effects of weight gain and restoration on BMD. However, a systematic review posing this issue as a primary outcome has not yet been conducted. Indeed, available reviews (critical, systematic or expertise) have considered this a marginal issue, and some, in any case, failed to meet the criteria for “homogeneity” (e.g., including both adolescent and adult populations) and “completeness” (e.g., including only a part of the available literature) [12,24]. In order to provide a less biased interpretation of the evidence to date on BMD, we decided to focus on adolescents, as bone metabolism seems to occur differently in these patients than in adults with AN [25]. We set out to systematically review the published literature on the topic in accordance with the PICO process, as detailed below [26]. To the best of our knowledge, this is the first such systematic review conducted on adolescents with AN.

P-Population: female and male adolescents who met the diagnosis criteria for AN and had an age range or mean age of between 10 and 19 years at baseline. I-Intervention: weight gain or weight restoration. C-Comparison: AN group before and after weight gain or weight restoration, and matched healthy control group (when available). O-Outcome: changes in total body and/or regional BMD however expressed (absolute value, *z*- or *t*-scores standard deviations, and increases in percentage from baseline to follow-up).

## 2. Experimental Section

Care was taken to adhere to the Preferred Reporting Items for Systematic Review and Meta-Analyses (PRISMA) guidelines in completion of this review [27].

### 2.1. Inclusion and Exclusion Criteria

All studies evaluating bone status in adolescents with AN were included, provided that they met the following criteria: (i) age range or mean age between 10 and 19 years [28]; (ii) written in English; (iii) original articles on studies with a longitudinal design; and (iv) prospective or retrospective observational (analytical or descriptive), experimental or quasi-experimental controlled or non-controlled studies documenting significant weight gain or weight restoration in patients with AN. No reviews, cross-sectional studies or non-original articles (i.e., case reports, editorials, Letters to the Editor, and book chapters) were included.

### 2.2. Information Source and Search Strategy

The literature search was designed and performed independently in duplicate by two authors. The PubMed database was systematically screened using the following MeSH terms: #1 anorexia nervosa, #2 adolescent, #3 weight gain, #4 weight restoration, #5 weight normalization, #6 bone disease, #7 bone mineral density, #8 peak bone mass, #9 osteoporosis, #10 osteopenia, and #11 bone mineral accrual. The following combinations were also applied as search parameters: (#1 AND #2 OR #3 OR #4 OR #5) AND (#6 OR #7 OR #8 OR #9 OR #10 OR #11), and a manual search was carried out to retrieve other articles that had not been identified via the initial search strategy. Publication date was not considered an exclusion criterion for the purposes of this review.

### 2.3. Study Selection

Two authors independently screened the resulting articles for their methodology and appropriateness for inclusion. Non-controlled studies were selected according to the National Institute for Health and Clinical Excellence (NICE) guidelines checklist for quality appraisal [29], in which a total score of 0–3 indicates poor quality; between 4 and 6, fair quality; and ≥7 good quality. In controlled studies, quality appraisal was conducted according to the Newcastle–Ottawa Scale (NOS) [30], which relies on a 9-star system in which scores of 0–3, 4–6, and 7–9 are considered poor, moderate and good quality, respectively. Scores of 4, 2 and 3 were, respectively, assigned to the “selection of study groups”, “comparability of study groups”, and “assessment of outcomes and adequacy of follow-up” criteria [31]. Consensus discussion was used to resolve disagreements between reviewers.

### 2.4. Data Collection Process and Data Items

First the, title and abstract of each paper were assessed by two independent authors for language suitability and subject matter relevance, and the studies thereby selected were assessed for their appropriateness for inclusion and quality of method. The first author, year of publication, design, sample age, baseline BMI, duration of illness (where available), duration of follow-up, site of BMD measurement, intervention, changes in BMD, and quality score of each study that passed these two rounds of screening are reported in Table 1.

### 2.5. Data Synthesis

Due to the lack of homogeneity among the resulting studies, a meta-analysis could not be performed. In particular, studies varied in terms of how improvements in BMD were measured (i.e., absolute values, *z*- and *t*-scores, and percentage increases), the DXA scan models assessed, and the sites at which measurement was performed. Hence this systematic review is presented as a narrative synthesis [32].

## 3. Results

**Figure 1:**
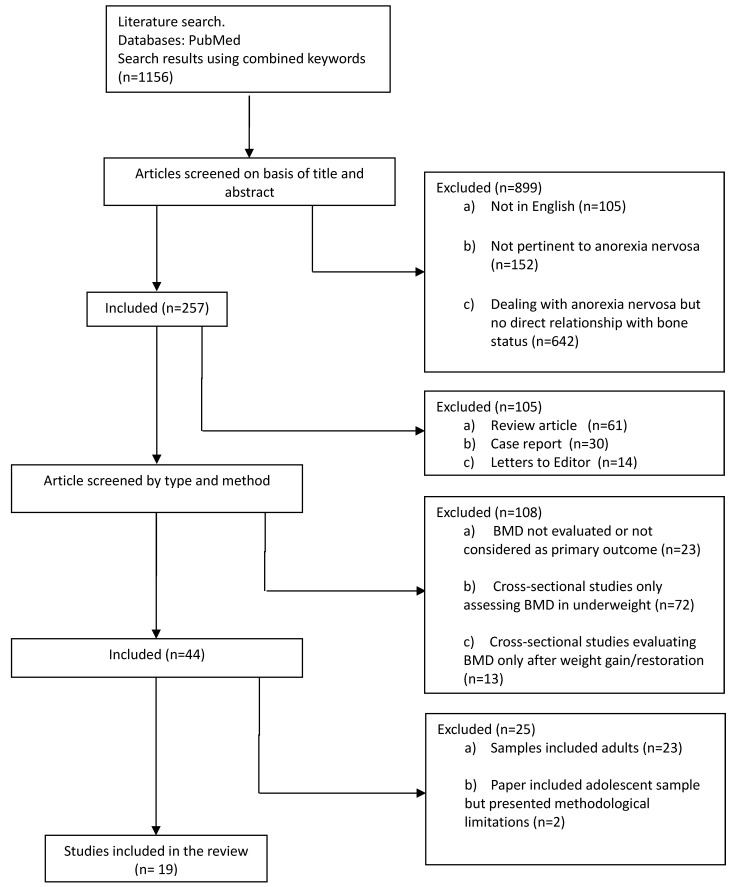
Flow chart summarizing the study selection procedure.

The initial search retrieved 1156 papers. After the first round of screening (titles and abstracts), 899 papers were excluded on the following grounds: 105 were in a language other than English; 152 had no bearing on AN; and 642 dealt with AN, but did not consider bone status. The second round of screening excluded review articles (*n* = 61), clinical case reports (*n* = 30) and Letters to the Editor (*n* = 14). Of the remaining 152 articles dealing with AN and bone status, a further 108 papers were excluded on the following grounds: failure to evaluate BMD or consider it as a primary outcome (*n* = 23) (i.e., bone turnover and hormonal aspects, bone geometry, bone strength and fracture risk, etc.); and cross-sectional designs that did measure change in BMD, i.e., assessing BMD either during underweight only (*n* = 72), or after weight gain or restoration (*n* = 13), but without reporting baseline measurements. As our review was to focus on adolescents, from the 44 papers evaluating changes in BMD measures before and after weight gain or restoration, we eliminated a further 23 papers because their samples included adult AN. Finally, of the 21 remaining papers, two were eliminated due to the following methodological limitations: (i) the BMD improvement achieved in the placebo arm group given only nutritional treatment was unclear; and (ii) both arms of patients were given osteotropic treatment (Figure 1).

Thus, 19 articles (15 non-controlled and four controlled studies) were available for systematic review and narrative analysis. According to the NICE guidelines checklist, the non-controlled studies (*n* = 15) were of fair quality (mean score 5.06 points) (see Table A1 in Appendix A), while the Newcastle–Ottawa Scale checklist indicated that the controlled studies (*n* = 4) were of moderate quality (mean score 6.25 points) (see Table A2 in Appendix A).

### 3.1. Studies in Adolescent Females with Anorexia Nervosa

#### 3.1.1. No Significant Change in BMD after Weight Gain/Restoration

Eight prospective studies (five non-controlled and three controlled) addressed this issue; six of those had a standardized follow-up period of 12 months, whereas two studies had non-standardized follow-up periods of 14.1 ± 5.4 and 19.4 ± 5.6 months, respectively. The overall quality of these studies was judged as fair–moderate.

In 1996, Kooh et al. [33] used dual energy X-ray absorptiometry (DXA) to measure lumbar spine (L1–L4) and femoral neck BMD in a prospective controlled study of 22 adolescent females with AN and 24 aged-matched healthy controls. After a non-standardized period of follow-up, which varied between 7 and 26 months, 12 out of the 22 patients had gained significant weight (mean weight gain = 4.9 kg), but showed no change in BMD with respect to baseline. There was also no apparent improvement in BMD in the four patients who achieved weight restoration (BMI > 20 kg/m^2^) and resumed menstruation.

In 2002, Muňoz et al. [19] also failed to find an association between weight gain and change in lumbar BMD in a prospective non-controlled DXA study based on the normative data of a standard Spanish population of 38 adolescent (between 15 and 20 years old) females with AN. Despite their reportedly significant weight gain (although clear statistics are unavailable), no change in the lumbar spine (L2–L4) BMD, expressed as a *z*-score, was seen in the 12 patients who completed the 12-month follow-up.

In 2002, another prospective non-controlled DXA study, conducted by Golden et al. [34], measured BMD at the lumbar spine (L2–L4) and femoral neck in 28 adolescent females with AN over a mean follow-up period of 23.1 ± 11.4 months. Although 12-month follow-up data on changes in body weight and BMD were only available in 25 patients, these had gained significant weight (mean weight gain = 7.1 ± 9.0 kg). Nevertheless, no improvement was seen in terms of absolute values for BMD at either the lumbar spine or the neck of the femur.

In 2002, Soyka et al. [35] conducted a prospective controlled study to assess bone mineral accrual rate in 19 patients with AN, and 19 healthy age-matched controls. DXA was used to measure BMD, and total body and lumbar spine (L1–L4) bone mineral accrual rates were expressed as the change from baseline to 12-month follow-up. In this study, 11 patients achieved weight restoration (BMI = 18.9 ± 0.6 kg/m^2^) during the assessment period, and although lumbar spine BMD increased in healthy controls, no changes were detected in girls with AN.

In 2006, Compston et al. [36] published a prospective non-controlled study in which they measured the regional BMD at the lumbar spine (L1–L4) and proximal femur via DXA, calculating *z*-scores with respect to those furnished by a reference DXA scan. Twenty-one patients completed the 12-month follow-up with a mean weight gain of ≈10 kg, but no significant changes in lumbar spine, femoral neck, total hip or total BMD were detected.

In 2007, Oświęcimska et al. [37] measured total body and lumbar spine BMD at baseline, in a prospective non-controlled study of 18 adolescent females with AN. After a non-standardized follow-up period (19.4 ± 5.6 months), in which the sample as a whole reached a BMI of 18.90 ± 2.91 kg/m^2^, nine of the 18 patients had achieved the BMI ≥ 18.5 kg/m^2^ threshold and resumed menstruation. However, despite maintenance of normal weight, DXA showed no significant changes in the mean values for total body and lumbar spine BMD. Furthermore, patients failed to show the expected age-related increase in BMD during follow-up.

In a larger prospective controlled study, published in 2008, Misra et al. [38] compared lumbar (L1–L4) and whole body BMD in 34 adolescent females with AN and 33 controls, matched by height, and chronological, skeletal and menarchal ages. By 12-month follow-up, 14 patients had achieved weight restoration (ΔBMI = +3.14 kg/m^2^) and resumption of menses. However, according to DXA, this was associated with merely a stabilization of BMD measures.

In 2014, Franzoni et al. [39] used DXA measurements to assess total body and lumbar spine (L1–L4) BMD in a prospective non-controlled study on 79 adolescent females with AN, reporting *z*-scores for lumbar BMD for the 46 patients who completed the 12 months of follow-up. After 12 months, patients showed a significant weight gain from baseline (ΔBMI = +1.29 ± 1.85 kg/m^2^), but no significant improvement in lumbar *z*-scores. However, patients who were involved in a competitive sport showed better bone status.

#### 3.1.2. Improvement/Normalization in BMD after Weight Gain/Restoration

Nine studies (five prospective non-controlled, three retrospective non-controlled and one prospective controlled) addressed this issue; eight of those had a standardized follow-up period, which varied between 12 and 90 months, and one study had a non-standardized follow-up period of 15.4 ± 6.1 months. The quality of the eight non-controlled studies was judged as fair, and the sole controlled study was judged to be of high quality.

In 1991, Bachrach et al. [40] conducted a prospective non-controlled study of BMD in 15 adolescent females with AN using dual photon absorptiometry of the lumbar spine (L2–L4). Twelve to 16 months from baseline, nine of the 15 patients had gained weight (between 4.7 and 17.4 kg), and showed significant improvements in whole body BMD (from 0.710 ± 0.118 at baseline to 0.773 ± 0.105 at follow-up), and seven of these showed increased lumbar spine BMD. Despite these improvements, osteopenia of the spine and/or whole body was still apparent when the mean standard deviations (SD) for normal adolescents were used as comparison.

In 2001, Jagielska et al. [41] also used DXA, in a non-controlled study to measure total body and lumbar spine (L2–L4) BMD in 42 adolescent females with AN. *Z*-scores (number of SDs above or below normal mean BMD values matched for age and sex) were assigned, and five densitometric assessments were performed from baseline over a 28-month follow-up period. Only 11 patients attended all five assessments, but all achieved a substantial weight gain. Only the fourth and fifth densitometric assessments (21 and 28 months, respectively) showed significant improvements in lumbar and total BMD values, from 1.010 ± 0.1 at baseline to 1.044 ± 0.099 at the fourth assessment, and from 1.030 ± 0.11 at baseline to 1.058 ± 0.11 at the fifth assessment, respectively.

In 2001, Castro et al. [42] used DXA to assess BMD in the lumbar spine (L2–L4) and the femoral neck in a prospective non-controlled study on 108 female patients with AN. These authors measured BMD at baseline and after a non-standardized follow-up period of between 6 and 30 months. Forty-four patients achieved weight restoration (BMI > 19 kg/m^2^), and those who displayed reduced BMD at baseline (*z*-score < −1) (*n* = 23) showed a significant increase in both the lumbar spine (+9.1% per year) and femoral neck (+4.5% per year) at follow-up.

In 2005, Bass et al. [43] published a retrospective non-controlled DXA study of total and lumbar spine BMD in 13 weight-restored, menstruation-resumed adolescent females with AN (weight within 15% of that expected for age and height), showing improvement in these parameters over the course of a 40-month follow-up period. After ≈30 months from recovery, BMD SD scores had increased by +1.5 SD at the lumbar spine, interpreted as near normalization, and +1.2 SD in the whole body, interpreted as complete normalization.

In 2005, another DXA study, originally designed to examine the effect of oral alendronate on bone loss, was conducted by Golden et al. [22]. The placebo arm (*n* = 15) of that study achieved a mean weight-gain percentage of 16.2% at 12-month follow-up, accompanied by an increase in volumetric BMD of 2.3% ± 6.9% in the femoral neck and 2.2% ± 6.1% in the lumbar spine. However, in spite of these improvements, the BMD remained low at 12 months, with fewer, than a third of patients having bone mass restored to the normal range.

However, another prospective non-controlled study, published in 2007 by Mika et al. [44], did find small improvements in BMD. They used DXA to measure lumbar (L2–L4) and femoral neck BMD in 19 adolescent females with AN, who all gained weight during a 15-week inpatient treatment program. Ten out of the 19 patients had maintained the restored weight (BMI ≥ 10th percentile) at two-year follow-up, and showed improvements in lumbar (Δ BMD = +0.077 ± 0.042) and femur BMD (Δ BMD = +0.057 ± 0.044).

In 2007, do Carmo et al. [45] used DXA in a retrospective non-controlled assessment of changes in lumbar (L2–L4) and femoral neck BMD over 90 months of follow-up. In 15 adolescents with AN at baseline, BMI increased from 15.9 ± 4.8 kg/m^2^ to 20.6 ± 5.8 kg/m^2^ at follow-up, with 11 out of 15 patients achieving normal weight (BMI ≥ 18.5 kg/m^2^). These improvements were associated with positive changes in the mean lumbar (L2–L4) and femoral neck BMD in terms of *t*- (from −1.6 ± 1.2 and −0.9 ± 0.9 at baseline to −1.4 ± 1.2 and −1.0 ± 0.9, respectively) and *z*- (from −1.4 ± 1.1 and −1.1 ± 0.9 at baseline to −1.3 ± 1.2 and −1.0 ± 0.9, respectively) scores. However, despite weight restoration, six patients still showed signs of osteoporosis or osteopenia in the lumbar spine, and five had osteopenia in the femoral neck.

In 2010, Schulze et al. [46] reported their results of whole-body DXA scans performed in a retrospective non-controlled study of BMD in 52 adolescent females with AN, namely a significant improvement in total BMD after >36-months follow-up from baseline. This was associated with an increase in BMI across the whole sample, from 14.7 ± 1.9 kg/m^2^ to 20.1 ± 2.8 kg/m^2^. The post-treatment outcomes of 26 of the 52 patients were classed as good (BMI ≥ 17.5 kg/m^2^ and resumption of menses), with a mean BMI of 20.5 ± 2.2 kg/m^2^ at follow-up, and these showed a significant increase in total body BMD (ΔBMD = +0.08 ± 0.07). A gain in total body BMD also seemed to be correlated with regular physical activity.

In 2011, Misra et al. [47] conducted a randomized controlled study designed to evaluate the effect of estrogen replacement on BMD at the spine and hip in adolescent girls with AN. Their placebo arm, composed of 30 patients, showed a significant increase in weight (+4.18 ± 0.99 kg) and BMI (1.47 ± 0.36 kg/m^2^), at 18-month follow-up. According to DXA measurements, this was associated with an increase in lumbar BMD (+0.002 ± 0.011), and in percentage of lumbar BMD (+0.307% ± 1.144%). Nevertheless, it should be noted that the bone mineral accrual rate seen in these patients was lower than that in the normal-weight girls taken as control.

#### 3.1.3. Reduction in BMD after Weight Gain

In direct opposition to these findings, Stone et al. [48] found a significant decrease in age- and height-standardized total body BMD ((−0.81 ± 0.63; −0.88 ± 1.07 for age) and (−0.23 ± 0.47; −0.34 ± 0.72 for height) in premenarchal and postmenarchal subjects, respectively)), femoral neck BMD ((−0.87 ± 0.75; −0.67 ± 0.75 for age) and (−0.61 ± 0.72; −0.51 ± 0.75 for height) in premenarchal and postmenarchal subjects, respectively)) and lumbar spine BMD ((−0.67 ± 0.46; −0.97 ± 0.70 for age) and (−0.22 ± 0.34; −0.60 ± 0.48 for height) in premenarchal and postmenarchal subjects, respectively)) over 12 months of assessment. However, although DXA was used to measure total body, femoral neck and lumbar (L2–L4) BMD in 30 adolescent females with AN who had gained significant weight (+19% in premenarchal and +5.6% in postmenarchal subjects) between baseline and follow-up, this data derived from a single, non-controlled retrospective study judged to be of poor quality.

### 3.2. Studies in Adolescent Males with Anorexia Nervosa

In one prospective non-controlled study dated 2002, judged to be of fair quality, Castro et al. [10] used DXA to measure BMD at the lumbar spine (L2–L4) and the femoral neck in 20 adolescent males with AN. Fifteen patients completed a non-standardized follow-up period, which ranged between 6 and 24 months. Interestingly, although weight-restored patients (BMI ≥ 19 kg/m^2^ (*n* = 9)) showed a significant increase in BMD from baseline of +7.8% per year at the lumbar spine and +6.7% per year at the femoral neck, the patients in the group that merely gained weight (BMI < 19 kg/m^2^ (*n* = 6)) showed a further BMD loss of −3.2% per year at the lumbar spine and −6.4% per year at the femoral neck. The level of physical activity was not an independent predictor of changes in BMD in either of these groups.

## 4. Discussion

### 4.1. Summary of Evidence

The findings of the studies included in this review can be classified as follows:

#### 4.1.1. Strong Evidence

Weight gain and/or weight restoration are associated with stabilization of BMD during the first year of follow-up in female adolescents with AN, as observed by six studies, judged objectively to be of fair–moderate quality, that featured a 12-month follow-up [19,34,35,36,38,39]. Although one study found contrasting findings, it was judged to be of poor quality [48].

Weight gain and/or restoration are associated with a significant increase in BMD after a year, as reported for most of the studies with a follow-up longer than 12 months [40,41,44,45,46,47]. This finding confirms the hypothesis that BMD gain is a slow process in adolescent females with AN, and longer time frames are required to detect improvements.

In our opinion, neither of these two findings requires further replication.

#### 4.1.2. Weak Evidence and Evidence Still Requiring Confirmation

According to one study, significant weight gain could be associated with a further reduction of BMD during the first 12 months of follow-up [48]. However, this finding can be classed as weak for at least two reasons: (i) according to the NICE guidelines checklist, this study was judged to be of poor quality; and (ii) the findings of this single study contrast with those falling into the “strong evidence” category.

One study found that normalization of total body and lumbar spine BMD is achievable within 30 months in female adolescents with AN after complete weight restoration (weight within 15% of that expected for age and height) and menses resumption [43]. Although still requiring confirmation, this finding is in line with the strong evidence described above, and underlines the reinforcement effect of “long-term follow-up” plus “normal weight maintenance”.

The sole-study finding that adolescent males with AN who gain significant weight but remain underweight experience a further loss in BMD, while those who achieve weight restoration have a significant increase in BMD and a bone accrual rate which doubles that seen in healthy male adolescents [10], also requires confirmation via long-term controlled studies on large samples.

### 4.2. Summary of Limitations

These findings should be interpreted with caution, bearing in mind the following limitations:

A small sample size was a common feature of the majority of the studies reviewed, particularly those involving male patients with AN. In fact, in 12 out of 19 studies, the number of participants who completed the follow-up assessments did not exceed 20 [10,19,22,33,35,37,38,40,41,43,44,45]. The samples in the other seven studies numbered 20 to 50 participants completing follow-up assessments [34,36,39,42,46,47,48].

Weight restoration was achieved in only nine out of 19 studies, which also featured heterogeneous definitions of normal-weight cut-offs (BMI ≥ 17.5 kg/m^2^, BMI ≥ 18.5 kg/m^2^, BMI ≥ 19.0 kg/m^2^ and BMI percentile ≥10th percentile) [10,35,37,38,42,43,44,45,46]. Furthermore, although weight restoration is one of the most important “physical” criteria of AN recovery, the absence of data on eating disorder psychopathology does not enable us to establish whether or not participants had, in fact, achieved complete “psychological” recovery.

Fifteen out of 19 studies had a non-controlled design [10,19,22,34,36,37,39,40,41,42,43,44,45,46,48]. They are therefore unable to establish whether patients with AN after weight gain and/or restoration achieved normal BMD measures as compared to matched healthy controls. Moreover, in only three out of four controlled studies [35,38,47], were the changes in BMD assessed in a matched healthy control group, although such evaluation is vital for determining the difference in bone accrual rates between patients from these two groups, and therefore to detect the BMD catch-up process.

Likewise, only three out of 19 studies assessed the relationship between the physical activity and changes in BMD [10,39,46], and four studies were retrospective [43,45,46,48], a design with inherent methodological limitations that may bias interpretation of results. Moreover, four studies [10,33,37,42] had a non-standardized follow-up, which varied between 6 and 30 months. Accordingly, these studies revealed inconsistent findings, since their samples included patients who underwent follow-up assessment after a short period of time (i.e., 6–8 months), and others assessed after a longer follow-up (28–30 months).

No study took into account the rate of weight gain, or eating disorder behaviors (i.e., self-induced vomiting, excessive physical exercise, laxative and diuretic misuse and binge-eating episodes) that might have influenced BMD gain and restoration. Moreover, the non-randomized studies were plagued by potential confounders such as body composition (lean and fat masses), amenorrhea, hormonal (estrogen or testosterone) and vitamin D levels, and only rarely took into account the influence these could have on changes in BMD during weight gain and restoration. Furthermore, only one non-controlled study assessed BMD changes in male adolescents with AN after weight gain and restoration [10].

### 4.3. Clinical Implications

Despite the considerable body of research conducted on bone disease in AN populations, guidelines for the management of bone mass loss in adolescent patients are still not forthcoming. Available recommendations derive from non-evidence-based sources (clinical experience, personal opinion, internet medical forums, etc.), or guidelines drawn up for different populations (i.e., adults with AN, menopausal women, etc.).

To date, the most tested treatments (i.e., hormone replacement, oral contraceptive pill, DHEA and biphosphonates) in adolescents with AN have yielded modest or negligible improvements in BMD [12], and no data showing a beneficial effect on BMD of other strategies, such as physical activity interventions and/or nutritional supplementations (calcium, vitamin d, etc.) are available [12]. The only promising pharmacological treatment is physiological estrogen replacement by means of transdermal estradiol associated with cyclic progesterone, which, despite a similar weight gain (about 4 kg at 18 months), was associated with a significantly greater increase in spine and hip BMD than the placebo in non-severely underweight adolescents with AN (BMI 17.4 ± 0.1) [47]. However, these data require a replication in patients with more severe malnutrition.

Nevertheless, our systematic review provides benchmark data on the association between weight gain/restoration and BMD in adolescents females with AN, which can be used by directly (i.e., specialists of eating disorders) or indirectly involved (i.e., endocrinologists, rheumatologists, psychiatrists, gynecologists, gastroenterologists, etc.) clinicians to manage reduced BMD in these patients. The data reported above can be also used by clinicians to inform patients and/or their legal guardians (when under 18) that, although bone mass improvement and normalization is a very slow process, the best strategy to address reduced BMD is weight gain/restoration, which arrests any further BMD loss and results in BMD stabilization during the first year of follow-up. Improvements in BMD are not usually observed before 16 months from weight restoration, and normalization seems to occur only after ≈3 years of “normal weight maintenance” with resumption of regular menses.

## 5. Conclusions and Areas for Future Research

This systematic review of 19 studies judged objectively to be of fair–moderate quality has several clinical implications, some supported by strong evidence. Specifically, weight gain and/or weight restoration in adolescent females with AN is associated with BMD stabilization during the first year of follow-up [19,34,35,36,38,39], and significant improvements can be achieved in the long term (≈16-month follow-up) [40,41,44,45,46,47]. However, more research is needed to confirm the apparent association between long-term (30 months) normal-weight maintenance and menses resumption and 80% to 100% normalization of BMD in the lumbar spine and whole body, respectively [43]. Indeed, although the only other two studies with comparably long-term follow-up found an improvement in BMD, this did not reach normal values. That being said, in the do Carmo et al. [45] study, about 27% of patients had a BMI lower than 18.5 kg/m^2^, while in the sample studied by Schulze et al. [46], the threshold considered for normal BMI was 17.5 kg/m^2^.

It is also vital that more research is carried out on male adolescents with AN, in particular to confirm that if those who restore their weight (≥19 kg/m^2^) have a significant increase in BMD, and a bone accrual rate which may double that seen in healthy male adolescents [10]. In addition, the indication that if those who achieved weight gain but remain underweight experience a further loss in BMD merits further exploration [10].

As these latter findings were generally the fruit of single studies involving small populations, future research must seek to overcome such limitations. Standardized, well designed, prospective controlled studies with large samples and long-term follow-up are required to collect sufficient scientific information on the effects of “complete” weight restoration on BMD for meta-analysis, particularly in adolescent males with AN before and after weight restoration. Future studies should also attempt to answer several unresolved but important clinical questions regarding the age at which it is no longer possible to reverse the BMD loss associated with AN, the best strategies to optimize peak bone mass acquisition and BMD normalization in adolescents with AN (i.e., weight gain rates, physical activity interventions, pharmacotherapy, hormone replacements), and how to manage the loss of BMD in patients who are reluctant to gain weight.

## Figures and Tables

**Table 1:** Studies included in the systematic review.

First Author	Year	Study Design	Sample	Age	Baseline BMI	Duration of Illness	Follow-Up	Site	Intervention Outcome	Change in BMD	Quality Score
*A. Studies in adolescent females with anorexia nervosa*											
1. No significant change in BMD after weight gain/restoration											
Kooh et al. [33]	1996	Prospective controlled	*N* = 12 completers from an original sample of *n* = 22	14–21 years	15.9 ± 2.2 kg/m^2^	Not available	Non-standardized, between 7 and 26 months; Mean follow-up 14.1 ± 5.4 months	Lumbar spine; Femoral neck	Weight gain, mean 4.9 kg	No change in BMD	5 **
Muňoz et al. [19]	2002	Prospective non-controlled	*N* = 38; *N* = 12 completers	17.4 ± 1.5 years	−1.4 ± 0.5 SD	Not available	12 months	Lumbar spine (L2–L4)	Weight gain, expressed as SD BMI	No change in BMD from baseline to follow-up	5 *
Golden et al. [34]	2002	Prospective non-controlled	*N* = 28; *N* = 25 completers	13–21 years	16.9 ± 1.5 kg/m^2^	21.9 ± 20.6 months	12 months	Lumbar spine (L2–L4) and femoral neck	Weight gain, mean 7.1 ± 9.0 kg	No significant improvement in lumbar spine or femoral neck BMD from baseline to follow-up	6 *
Soyka et al. [35]	2002	Prospective controlled study	*N* = 19	12.9–17.8 years	16.4 ± 0.5 kg/m^2^	14.0 ± 3.0 months	12 months	Total body and lumbar BMD	Weight restoration, BMI = 18.9 ± 0.6 kg/m^2^ in 11 participants	Lumbar BMD remained lower than that in controls	6 **
Compston et al. [36]	2006	Prospective non-controlled	*N* = 26; *N* = 21 completers	13–20 years	14.2 ± 1.7 kg/m^2^	Not available	12 months	Lumbar spine and proximal femur BMD	Weight gain during treatment ~10 kg	No significant changes in BMD of lumbar spine, femoral neck, total hip or total body	7 *
Oświęcimska et al. [37]	2007	Prospective non-controlled	*N* = 18	11.5–18.1 years	15.8 ± 2.1 kg/m^2^	14.9 ± 13.6 months	Non-standardized, mean 19.4 ± 5.6 months	Total body and lumbar spine BMD	Weight restoration (BMI ≥ 18.5 kg/m^2^) and resumption of menstrual cycle in 9/18 patients	No significant changes in mean BMD of total body or lumbar spine; Significant reduction in total body BMD *z*-score at follow-up	6 *
Misra et al. [38]	2008	Prospective controlled	*N* = 34; *N* = 14	12–18 years	16.6 ± 1.2 kg/m^2^	11.2 ± 12.4 months	12 months	Lumbar and total BMD	Weight restoration	Stabilization of BMD but no improvement	7 **
Franzoni et al. [39]	2014	Prospective non-controlled	*N* = 79; *N* = 46 completers	11–22 years	16.3 ± 1.3 kg/m^2^	27.8 ± 23.9 months	12 months	Lumbar BMD	Weight gain (ΔBMI = +1.29 ± 1.85 kg/m^2^)	No significant changes in lumbar BMD *z*-score from baseline to one-year follow-up	5 *
2. Improvement/normalisation in BMD after weight gain/restoration											
Bachrach et al. [40]	1991	Prospective non-controlled	*N* = 15	16.7 ± 2.4 years	15.8 ± 1.7 kg/m^2^	Not available	12–16 months	Spine (L2–L4) and whole BMD	Weight gain of 4.7–17.4 kg in 9 patients	Increase in whole body BMD No changes in the spine Persistent osteopenia	4 *
Jagielska et al. [41]	2001	Prospective non-controlled	*N* = 42; *N* = 11 completers	10.8–22.2 years	14.7 ± 2.4 kg/m^2^	14.1 ± 17.4 months	28 months	Total and lumbar spine BMD as absolute value and *z*-score	Weight gain, from BMI 14.7 ± 5.4 kg/m^2^ at baseline to 19.8 ± 3.0 kg/m^2^	Increase in lumbar and total BMD after only 21 months of follow-up	5 *
Castro et al. [42]	2001	Prospective non-controlled	*N* = 108; *N* = 23	12–17 years	16.0 ± 1.1 kg/m^2^	10.0 ± 5.4 months	Non-standardized, between 6 and 30 months. Mean follow-up 15.4 ± 6.1 months	Lumbar spine (L2–L4) and femoral neck	Weight restoration, BMI > 19 kg/m^2^	Increase in both lumbar spine and femoral neck BMD. Normalization of BMD in 4 patients	5 *
Bass et al. [43]	2005	Retrospective non-controlled	*N* = 13	13.4–18 years	15.3 ± 0.8 kg/m^2^	19.0 months	40 months	Total and lumbar spine BMD	Weight restoration	Normalization of total body BMD. ~80% improvement in lumbar spine BMD	5 *
Golden et al. [22]	2005	Prospective non-controlled	*N* = 17; *N* = 15 completers	13–21 years	16.4 ± 1.3 kg/m^2^	34.7 ± 28.0 months	12 months	Lumbar (L1–L4) and femoral neck BMD	Weight gain during treatment, ~16.2%	Increase in lumbar and femoral neck BMD; Normalization in less than one-third of patients	5 *
Mika et al. [44]	2007	Prospective non-controlled	*N* = 19	Mean 14.4 ± 1.6 years	14.2 ± 1.4 kg/m^2^	10.6 ± 6.7 months	24 months	Lumbar and femoral neck BMD	Weight gain, and 10/19 patients maintained restored weight (BMI ≥ 10th percentile)	Small improvements in BMD of lumbar and femoral neck from baseline to follow-up	6 *
do Carmo et al. [45]	2007	Retrospective non-controlled	*N* = 68; *N* = 15 completers	13–19 years	15.1 ± 1.3 kg/m^2^	Not available	90 months	Total body, femoral neck and lumbar (L1–L4) BMD	Weight restoration and maintenance in 11/15 patients (BMI ≥ 18.5 kg/m^2^)	Increase in mean t- and z- BMD scores of the lumbar (L2–L4) and femoral neck.	4 *
Schulze et al. [46]	2010	Retrospective non-controlled	*N* = 52	10–19 years	14.7 ± 1.9 kg/m^2^	Not available	>36 months	Total body BMD	Weight restoration, BMI ≥ 17.5 kg/m^2^ in 26/52 participants	Significant increase in total body BMD (ΔBMD = +0.08 ± 0.07)	5 *
Misra et al. [47]	2011	Prospective controlled	*N* = 110; *N* = 30	Mean 16.5 ± 0.2 years	17.4 ± 0.9 kg/m^2^	Not available	18 months	Spine (L1–L4) and hip BMD	Weight gain	Increase in lumbar BMD, which remained lower than that in normal-weight control girls.	7 **
3. Reduction in BMD after weight gain											
Stone et al. [48]	2006	Retrospective non-controlled	*N* = 30	Mean 14.6 years	14.9 kg/m^2^	Not available	12 months	Total body, femoral neck and lumbar (L1–L4) BMD	Weight gain during treatment, ~19% in premenarchal subjects and ~5.6% in postmenarchal subjects	Further reduction in all BMD measures	3 *
*B. Studies in adolescent males with anorexia nervosa*											
Castro et al. [10]	2002	Prospective non-controlled	*N* = 20; *N* = 15 completers	12–17 years	16.2 ± 1.2 kg/m^2^	12.5 ± 6.4 months	Non-standardized, between 6 and 24 months	Lumbar spine (L2–L4) and femoral neck	Weight gain group, BMI < 19 kg/m^2^ (*N* = 6); Weight restoration group, BMI ≥ 19 kg/m^2^ (*N* = 9)	Further BMD loss of −3.2%/year at lumbar spine and −6.4%/year at femoral neck in weight gain group. BMD gain of +7.8%/year at lumbar spine and +6.7%/year at femoral neck in weight restoration group	5 *

BMD: bone mineral density. * NICE guidelines checklist: Yes = 1, No (not reported, not available) = 0; Total score, 8; ≤3, poor quality; 4–6, fair quality; ≥7, good quality. ** Newcastle–Ottawa Scale (NOS) for longitudinal case control studies. Yes = 1, No (not reported, not available) = 0; Studies with scores of 0–3, 4–6, 7–9 were considered as low, moderate and high quality, respectively.

## Appendix A

**Table A1:** Quality assessment of non-controlled studies (in online supporting material).

	Bachrach 1991 [40]	Jagielska 2001 [41]	Castro 2001 [42]	Muňoz 2002 [19]	Golden 2002 [34]	Castro 2002 [10]	Golden 2005 [22]	Bass 2005 [43]	Stone 2006 [48]	Compston 2006 [36]	Mika 2007 [44]	do Carmo 2007 [45]	Oświęcimsk 2007 [37]	Schulze 2010 [46]	Franzoni 2014 [39]
Case series collected in more than one centre, i.e., multi-centre study	0	0	0	0	0	0	0	0	0	0	0	0	0	0	0
Is the hypothesis/aim/objective of the study clearly described?	1	1	1	1	1	1	1	1	1	1	1	1	1	1	1
Are the inclusion and exclusion criteria (case definition) clearly reported?	0	0	1	0	1	0	1	1	0	1	0	0	1	0	1
Is there a clear definition of the outcomes reported?	1	1	1	1	1	1	1	1	1	1	1	1	1	1	1
Were data collected prospectively?	0	1	1	1	1	1	1	0	0	1	1	0	1	0	1
Is there an explicit statement that patients were recruited consecutively?	0	0	0	0	0	0	0	0	0	1	1	0	0	1	0
Are the main findings of the study clearly described?	1	1	0	1	1	1	1	1	1	1	1	1	1	1	1
Are outcomes stratified? (e.g., by disease stage, abnormal test results, patient characteristics)	1	1	1	1	1	1	0	1	0	1	1	1	1	1	0
Total Score	4	5	5	5	6	5	5	5	3	7	6	4	6	5	5

NICE guidelines checklist: Yes = 1, No (not reported, not available) = 0; Total score, 8; ≤3, poor quality; 4–6, fair quality; ≥7, good quality.

**Table A2:** Quality assessment of controlled studies (in online supporting material).

Author	Kooh 1996 [33]	Soyka 2002 [35]	Misra 2008 [38]	Misra 2011 [47]
Selection				
Represents cases with independent validation	1	1	1	1
Cases are consecutive or obviously representative	1	0	0	0
Controls are from community	1	1	1	1
Controls have no history of Anorexia Nervosa	1	1	1	1
Comparability				
Controls are comparable for the most important factors.	1	1	1	1
Control for any additional factor	0	0	1	1
Ascertainment of exposure				
Secured record or structured interview where blind to case/control status	0	0	0	0
Same method of ascertainment for cases and controls	0	1	1	1
Cases and controls have completed follow up	0	1	1	1
Total score	5	6	7	7

Newcastle-Ottawa Scale (NOS) for longitudinal case control studies. Yes = 1, No (not reported, not available) = 0; Studies with scores of 0–3, 4–6, 7–9 were considered as low, moderate and high quality, respectively.
